# Supplementary material for: Proton transfer pathway in anion channelrhodopsin-1
Source: eLife. 2021 Dec 21;10:e72264. doi: 10.7554/eLife.72264 (PMC8691836; doi:10.7554/eLife.72264)
Supplement: Supplementary file 1. — (A) Calculated pKa values of Glu68 and Asp234 (PDB codes 6EDQ [Li et al., 2019] and 6CSM [Kim et al., 2018]). (B) Calculated pKa values of Glu68 and Asp234 in wild-type GtACR1 using the 10 MD-generated conformations with deprotonated Glu68/protonated Asp234. (C) Absorption wavelengths of 10 MD-generated structures calculated using Equation 1 with ΔEHOMO-LUMO (λHOMO-LUMO) and Equation 2 with ETD-DFT (λTD-DFT) (nm). (D) Calculated absorption wavelengths of wild-type GtACR1 with protonated Asp234 and D234E GtACR1 with protonated Glu234 (nm). The absorption wavelengths were calculated using Equation 1 with ΔEHOMO-LUMO (λHOMO-LUMO) and Equation 2 with ETD-DFT (λTD-DFT). Δλ234 (nm) denotes the electrostatic contributions of protonated Asp234 in wild-type GtACR1 and protonated Glu234 in D234E GtACR1 to the absorption wavelengths. (E) Binding energies between Cl– and the surrounding environments in Cl–-binding wild-type and E68Q/D234N mutant structures (kcal/mol). The binding energies were calculated based on the QM/MM-optimized and MD-based structures, using the MOE program (Molecular Operating Environment (MOE), 2021). (F) Calculated pKa values of Glu68 and Asp234 in wild-type GtACR1 using the 10 MD-generated conformations with protonated Glu68/deprotonated Asp234. (G) Absorption wavelengths of 10 MD-generated wild-type GtACR1 structures calculated using Equation 1 with ΔEHOMO-LUMO (λHOMO-LUMO) and Equation 2 with ETD-DFT (λTD-DFT) (nm). [file elife-72264-supp1.docx]

**Supplementary file 1A.** Calculated p*K*_a_ values of Glu68 and Asp234 (PDB codes 6EDQ ^1^ and 6CSM ^2^).

|  | **6EDQ** | **6CSM** |
| --- | --- | --- |
| Glu68 | 12 | 9.8 |
| Asp234 | –4.9 | –4.3 |

**Supplementary file 1B.** Calculated p*K*_a_ values of Glu68 and Asp234 in wild type *Gt*ACR1 using the 10 MD-generated conformations with deprotonated Glu68/protonated Asp234.

| **No.** | **Glu68** | **Asp234** |
| --- | --- | --- |
| 1 | 8.1 | 5.9 |
| 2 | 8.3 | 5.7 |
| 3 | 8.1 | 5.9 |
| 4 | 8.9 | 5.1 |
| 5 | 10.0 | 3.9 |
| 6 | 8.2 | 5.8 |
| 7 | 9.8 | 4.2 |
| 8 | 9.2 | 4.8 |
| 9 | 8.9 | 5.1 |
| 10 | 8.9 | 5.1 |

**Supplementary file 1C.** Absorption wavelengths of 10 MD-generated structures calculated using eq. 1 with Δ*E*_HOMO-LUMO_ (*λ*_HOMO-LUMO_) and eq. 2 with *E*_TD-DFT_ (*λ*_TD-DFT_) (nm).

|  | **wild type** | | **E68D** | | **E68Q** | |
| --- | --- | --- | --- | --- | --- | --- |
| No. | *λ*_HOMO-LUMO_ | *λ*_TD-DFT_ | *λ*_HOMO-LUMO_ | *λ*_TD-DFT_ | *λ*_HOMO-LUMO_ | *λ*_TD-DFT_ |
| 1 | 505 | 495 | 517 | 502 | 508 | 498 |
| 2 | 506 | 495 | 492 | 476 | 502 | 490 |
| 3 | 497 | 486 | 495 | 481 | 506 | 496 |
| 4 | 500 | 488 | 491 | 473 | 504 | 489 |
| 5 | 504 | 492 | 516 | 501 | 511 | 503 |
| 6 | 513 | 501 | 499 | 483 | 505 | 495 |
| 7 | 507 | 496 | 508 | 493 | 509 | 498 |
| 8 | 508 | 495 | 484 | 465 | 501 | 487 |
| 9 | 507 | 495 | 498 | 486 | 511 | 495 |
| 10 | 500 | 486 | 488 | 467 | 497 | 483 |

|  | **D234E** | | **D234N**  **(deprotonated E68)** | | **D234N**  **(protonated E68)** | |
| --- | --- | --- | --- | --- | --- | --- |
| No. | *λ*_HOMO-LUMO_ | *λ*_TD-DFT_ | *λ*_HOMO-LUMO_ | *λ*_TD-DFT_ | *λ*_HOMO-LUMO_ | *λ*_TD-DFT_ |
| 1 | 495 | 477 | 511 | 494 | 536 | 517 |
| 2 | 496 | 475 | 509 | 493 | 542 | 524 |
| 3 | 503 | 488 | 503 | 483 | 544 | 528 |
| 4 | 497 | 481 | 506 | 485 | 532 | 510 |
| 5 | 493 | 474 | 497 | 476 | 534 | 515 |
| 6 | 500 | 480 | 506 | 486 | 535 | 513 |
| 7 | 510 | 492 | 501 | 484 | 526 | 510 |
| 8 | 500 | 483 | 513 | 493 | 530 | 515 |
| 9 | 501 | 484 | 506 | 483 | 523 | 509 |
| 10 | 497 | 478 | 518 | 497 | 542 | 526 |

|  | **E68Q/D234N** | | **E68Q/D234N**  **(with Cl^–^)** | |
| --- | --- | --- | --- | --- |
| No. | *λ*_HOMO-LUMO_ | *λ*_TD-DFT_ | *λ*_HOMO-LUMO_ | *λ*_TD-DFT_ |
| 1 | 529 | 515 | 505 | 487 |
| 2 | 532 | 509 | 509 | 496 |
| 3 | 533 | 515 | 512 | 489 |
| 4 | 544 | 528 | 505 | 487 |
| 5 | 534 | 516 | 509 | 493 |
| 6 | 537 | 523 | 506 | 491 |
| 7 | 541 | 523 | 507 | 491 |
| 8 | 545 | 527 | 515 | 495 |
| 9 | 543 | 536 | 512 | 493 |
| 10 | 543 | 527 | 508 | 485 |

**Supplementary file 1D.** Calculated absorption wavelengths of wild type *Gt*ACR1 with protonated Asp234 and D234E *Gt*ACR1 with protonated Glu234 (nm). The absorption wavelengths were calculated using eq. 1 with Δ*E*_HOMO-LUMO_ (*λ*_HOMO-LUMO_) and eq. 2 with *E*_TD-DFT_ (*λ*_TD-DFT_). Δ*λ*_234_ (nm) denotes the electrostatic contributions of protonated Asp234 in wild type *Gt*ACR1 and protonated Glu234 in D234E *Gt*ACR1 to the absorption wavelengths.

|  | ***λ*_HOMO-LUMO_ ^a^** | ***λ*_TD-DFT_ ^a^** | **Δ*λ*_234_ ^a^** |
| --- | --- | --- | --- |
| wild type  (protonated D234) | 534 | 517 | –4 |
| D234E  (protonated E234) | 539 | 515 | –1 |

^a^ Average values of 10 QM/MM-optimized structures obtained after MD-simulations.

**Supplementary file 1E.** Binding energies between Cl^–^ and the surrounding environments in Cl^–^-binding wild-type and E68Q/D234N mutant structures (kcal/mol). The binding energies were calculated based on the QM/MM-optimized and MD-based structures, using the MOE program.

|  | **wild-type** | **E68Q/D234N** | **E68/D234N (MD-based)** |
| --- | --- | --- | --- |
| Thr71 | –3.9 | –3.8 | – |
| Ser97 | – | – | –4.1 |
| Asn234 | – | –7.7 | – |
| Lys238 (Schiff base) | –2.9 | –1.9 | –12.7 |
| H_2_O-507 | – | –3.0 | –3.5 |
| Total | –6.8 | –16.4 | –20.3 |

**Supplementary file 1F.** Calculated p*K*_a_ values of Glu68 and Asp234 in wild type *Gt*ACR1 using the 10 MD-generated conformations with protonated Glu68/deprotonated Asp234.

|  | **without POPC ^a^** | | **with POPC ^b^** | |
| --- | --- | --- | --- | --- |
| No. | **Glu68** | **Asp234** | **Glu68** | **Asp234** |
| 1 | 15 | –3.4 | 15 | –4.7 |
| 2 | 16 | –3.2 | 17 | –4.5 |
| 3 | 15 | –4.8 | 16 | –6.1 |
| 4 | 16 | –4.7 | 18 | –5.3 |
| 5 | 15 | –3.3 | 16 | –4.3 |
| 6 | 15 | –4.6 | 17 | –5.3 |
| 7 | 17 | –2.9 | 18 | –3.8 |
| 8 | 14 | –2.8 | 16 | –3.6 |
| 9 | 15 | –4.0 | 17 | –4.7 |
| 10 | 15 | –3.5 | 17 | –4.7 |

^a^ Calculated in the absence of POPC molecules. ^b^ Calculated in the presence of POPC molecules.

**Supplementary file 1G.** Absorption wavelengths of 10 MD-generated wild type *Gt*ACR1 structures calculated using eq. 1 with Δ*E*_HOMO-LUMO_ (*λ*_HOMO-LUMO_) and eq. 2 with *E*_TD-DFT_ (*λ*_TD-DFT_) (nm).

|  | **without POPC ^a^** | | **with POPC ^b^** | |
| --- | --- | --- | --- | --- |
| No. | *λ*_HOMO-LUMO_ | *λ*_TD-DFT_ | *λ*_HOMO-LUMO_ | *λ*_TD-DFT_ |
| 1 | 505 | 495 | 505 | 493 |
| 2 | 506 | 495 | 506 | 490 |
| 3 | 497 | 486 | 499 | 485 |
| 4 | 500 | 488 | 498 | 486 |
| 5 | 504 | 492 | 504 | 491 |
| 6 | 513 | 501 | 514 | 499 |
| 7 | 507 | 496 | 508 | 491 |
| 8 | 508 | 495 | 508 | 491 |
| 9 | 507 | 495 | 506 | 492 |
| 10 | 500 | 486 | 498 | 483 |
| average | 505 | 493 | 505 | 490 |

^a^ Calculated in the absence of POPC molecules. ^b^ Calculated in the presence of POPC molecules.
